# Integrated Approaches in the Management of Gastrointestinal Disorders: A Biopsychosocial Perspective

**DOI:** 10.7759/cureus.60962

**Published:** 2024-05-23

**Authors:** Muhammad Aamir Chughtai, Munara K Kerimkulova, Omid Mushtaq, Vibhavari Hagenahalli Anand, Abdur Rehman, Abdullah Shehryar, Baran Hassan, Rabia Islam, Hamza Islam, Muzafar Mansoor, Shehryar Rehman

**Affiliations:** 1 Internal Medicine, Punjab Medical College, Faisalabad, PAK; 2 Internal Medicine, Kyrgyz State Medical Academy, Bishkek, KGZ; 3 Preventive Medicine, Sakarya University Education and Research Hospital, Sakarya, TUR; 4 Critical Care Medicine, Aster RV Hospital, Bangalore, IND; 5 Surgery, Mayo Hospital, Lahore, PAK; 6 Internal Medicine, Allama Iqbal Medical College, Lahore, PAK; 7 Internal Medicine, College of Medicine, Hawler Medical University, Erbil, IRQ; 8 Research, Punjab Medical College, Faisalabad, PAK; 9 Internal Medicine, Al Assad University Hospital, Damascus, SYR

**Keywords:** lifestyle factors & ibd, treatment of ibs, ibd associated cancer, ibs subtypes, prevalence of gerd

## Abstract

Gastrointestinal (GI) disorders, including gastroesophageal reflux disease (GERD), inflammatory bowel disease (IBD), gastritis/peptic ulcer disease (PUD), and celiac disease, significantly impact global health and economic stability. This review synthesizes current literature to elucidate the pathophysiology, clinical manifestations, diagnostic challenges, and management strategies of these prevalent conditions. Through a biopsychosocial lens, we examine the role of the gut microbiome in disease modulation and explore innovative therapeutic advancements, including microbiome-targeting interventions. The review highlights the necessity of a multidisciplinary approach to patient care, integrating medical treatment with dietary, psychological, and lifestyle modifications. By addressing these disorders holistically, the article aims to foster a deeper understanding of their biopsychosocial impacts and encourage more effective, patient-centered treatment paradigms. The findings underscore the imperative for continued research and interdisciplinary collaboration to enhance patient outcomes and reduce healthcare burdens associated with GI disorders.

## Introduction and background

Gastrointestinal (GI) conditions are highly prevalent, disruptive illnesses spanning structural abnormalities like diverticula, inflammatory disorders like inflammatory bowel disease (IBD), disturbances of gut microbiota homeostasis seen in antibiotic-associated diarrhea, and functional GI issues manifesting in dysmotility or visceral hypersensitivity syndromes like irritable bowel syndrome (IBS). These heterogeneous diseases share common symptoms of abdominal pain, nausea, vomiting, diarrhea, or constipation, sometimes chronically. Quality of life is often significantly impaired with substantial indirect costs beyond direct medical expenses. 

In the United States, over 70 million ambulatory care visits each year stem from GI complaints, resulting in around 113 million days of restricted activity annually [1]. The collective economic burden is estimated at $142 billion when considering medical, hospital, and drug costs coupled with productivity losses [2]. Mental health comorbidities like anxiety and depression further compound disability, especially in persistent functional GI disorders. Hence GI diseases inflict immense health and societal burdens. 

This review synthesizes recent literature surrounding four highly impactful classes of digestive disturbances, gastroesophageal reflux disease (GERD), IBD, gastritis/peptic ulcer disease (PUD), and celiac disease. It elucidates pathophysiologic underpinnings, diagnostic challenges, management strategies, quality of life impacts, and directions for future therapies. By spotlighting these “big four” GI culprits through an expansive biopsychosocial lens, this article informs a nuanced, holistic understanding of alleviating suffering in those battling digestive dilemmas.

## Review

Gastroesophageal reflux disease

Pathophysiology of Gastroesophageal Reflux Disease

GERD is characterized by the frequent backflow of stomach contents into the esophagus, primarily due to a dysfunction of the lower esophageal sphincter (LES). This backflow results from the LES's inability to maintain a barrier between the stomach and esophagus, exacerbated by conditions such as hiatal hernia and delayed gastric emptying. The refluxed gastric contents, which are highly acidic, irritate and damage the esophageal epithelial tissue, leading to a range of symptomatic expressions [3]. Factors that diminish esophageal clearance and mucosal resistance also contribute to the severity of GERD, influencing the frequency and intensity of reflux episodes and the potential for tissue damage.

Clinical Manifestations and Complications

The clinical presentation of GERD is dominated by heartburn and regurgitation, the characteristic symptoms of the disease. However, the spectrum of manifestations includes erosive esophagitis, which indicates significant tissue injury and raises the risk of complications such as bleeding and esophageal strictures. A particularly severe complication is Barrett’s esophagus, a condition in which normal esophageal epithelium transforms into a metaplastic columnar epithelium, increasing the risk for dysplasia and esophageal adenocarcinoma. Additionally, GERD can cause extraesophageal symptoms such as chronic cough, laryngitis, and asthma, which may not immediately be recognized as linked to reflux [4].

Diagnostic Approaches

Diagnosing GERD typically begins with a clinical evaluation of symptoms, supplemented by a detailed patient history that guides further diagnostic testing. Endoscopic examination allows direct visualization of the esophageal mucosa and assessment of damage severity. Ambulatory reflux monitoring, including pH and impedance tests, quantifies acid exposure and reflux frequency, providing objective evidence to confirm the diagnosis. Symptom questionnaires can help correlate subjective symptoms with reflux events. Moreover, a therapeutic trial of proton pump inhibitors (PPIs) is often employed; a positive response can be a supportive diagnostic indicator [5].

Management and Treatment Options

**Figure 1:**
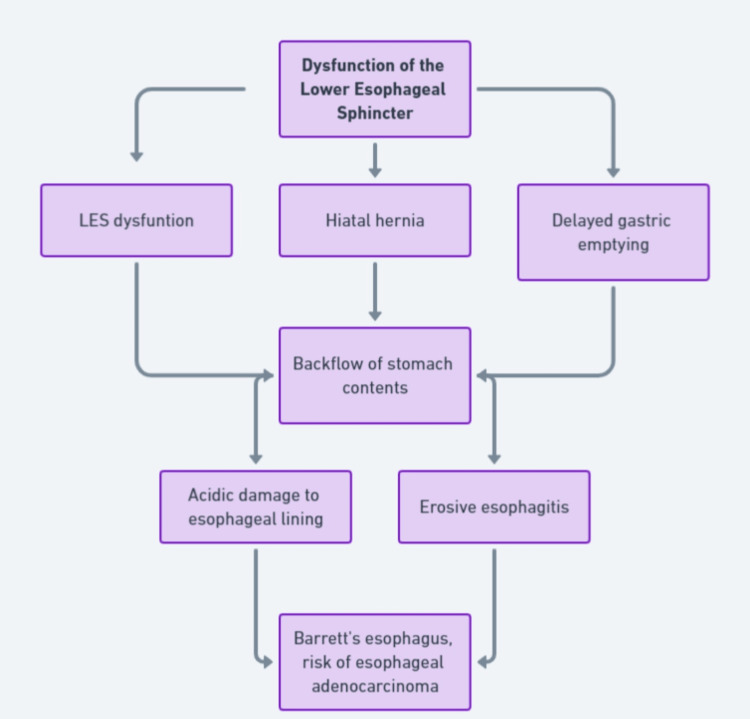
Flowchart depicting the pathophysiology of GERD. The image is created by the authors. GERD, gastroesophageal reflux disease

Management strategies for GERD aim to reduce acid exposure, improve esophageal clearance, enhance mucosal defenses, and alleviate symptoms. Lifestyle modifications such as dietary changes, weight loss, and elevating the head of the bed are recommended for all patients. Pharmacologic treatment primarily involves PPIs, which are the most effective in reducing gastric acid secretion. Histamine receptor blockers and prokinetic agents are also used. For patients with refractory symptoms, anti-reflux surgery, such as Nissen fundoplication, may be considered. Additionally, less invasive options such as transoral incisionless fundoplication are becoming more prevalent. Complementary approaches, including dietary supplements and herbal remedies, may provide symptomatic relief and are increasingly considered valuable adjuncts to standard medical treatments [6,7]. A flowchart depicting the pathophysiology of GERD is given in Figure 1.

Inflammatory bowel disease: Crohn’s and ulcerative colitis

Understanding Inflammatory Bowel Disease: Crohn’s Disease and Ulcerative Colitis

IBD represents a group of chronic, progressive inflammatory conditions affecting the GI tract, primarily characterized by intermittent flares and remission phases. Crohn’s disease can affect any part of the GI tract from mouth to anus and is noted for its transmural inflammation, which can lead to complications such as fistulas and strictures. Conversely, ulcerative colitis is confined to the colonic mucosa and often presents with continuous lesions. The pathogenesis of both conditions involves a complex interplay of genetic predispositions and immune system responses that are improperly regulated, often triggered by environmental and microbial factors [8].

Etiology and Risk Factors

The etiology of IBD is multifactorial, involving a significant genetic component where multiple genes contribute to increased inflammation and immune system dysfunction. Environmental factors, such as diet, smoking, and hygiene, have been shown to influence the incidence and course of the disease. Smoking, for example, is known to exacerbate Crohn’s Disease but may paradoxically have a protective effect in Ulcerative Colitis. Additionally, alterations in the gut microbiome play a crucial role, with dysbiosis contributing to the pathogenic processes by disturbing mucosal immunity and barrier functions [9].

Symptoms, Diagnosis, and Monitoring

Symptomatically, both Crohn’s and Ulcerative Colitis manifest with abdominal pain, severe bloody diarrhea, and weight loss. Fatigue is also common, significantly impacting the quality of life. Diagnosis primarily relies on endoscopic examination supplemented by histological analysis of biopsy samples, which help distinguish between Crohn’s and ulcerative colitis based on the pattern of inflammation observed. Non-invasive tests such as fecal calprotectin are useful for monitoring disease activity and response to treatment. Imaging studies, including CT and MRI, play crucial roles in assessing disease extent and complications [10].

Therapeutic Strategies and Patient Management

**Figure 2:**
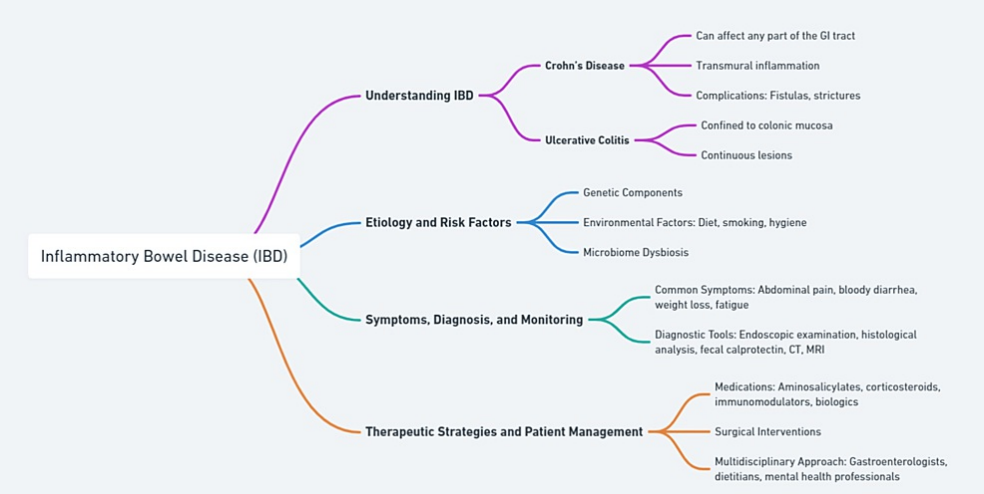
Mind map for IBD. The image is created by the authors. IBD, inflammatory bowel disease

Treatment of IBD aims to reduce inflammation, maintain remission, and prevent complications. The therapeutic arsenal includes aminosalicylates for mild to moderate disease, corticosteroids for acute flares, and immunomodulators and biologics for more severe cases. The choice of medication depends on disease severity, location, and response to previous treatments. Surgical interventions might be necessary for managing complications or disease refractory to medical treatment. A multidisciplinary approach involving gastroenterologists, dietitians, and mental health professionals is crucial for addressing the comprehensive needs of IBD patients, focusing on both physical symptoms and overall well-being [11,12]. A mind map for IBD is given in Figure 2.

Gastritis: a common yet complex condition

Types and Causes of Gastritis

Gastritis, or stomach lining inflammation, has numerous potential causes, most commonly NSAID injury, *Helicobacter pylori* infection, and autoimmune processes [13]. Variants include acute (sudden), chronic (persistent), erosive (damaging), atrophic (thinning mucosa), and special forms like eosinophilic or lymphocytic gastritis with distinct immune cells infiltrating tissue.

Symptoms and Diagnostic Tests

Epigastric pain, nausea, vomiting, bloating, or blood in emesis can indicate gastritis. Endoscopy visualizes tissue damage and acquires biopsies checking for *H. pylori* or unusual cell types while laboratory assays identify antibodies and vitamin/mineral deficiencies stemming from impaired absorption [14].

Treatment Approaches

Addressing the root cause is key, whether antibiotics for *H. pylori*, immune modulators for autoimmune gastritis, or eliminating triggering foods/medications [15]. Protecting the stomach lining supports healing, using acid suppression, mucosa coaters, and antioxidants.

Lifestyle and Dietary Modifications

Lifestyle adjustments to reduce gastritis flares include avoiding offending substances (alcohol, tobacco, and NSAIDs), reducing stress, adding probiotics, and eating smaller meals more frequently [16]. Dietary components like vitamin C, zinc, and plant polyphenols support the intestinal barrier and immune resilience.

Celiac disease: beyond gluten intolerance

The Immunology of Celiac Disease

Celiac disease is a complex autoimmune condition triggered by the ingestion of gluten in genetically susceptible individuals. Upon exposure to gluten, specific peptides stimulate an immune response in the small intestine, leading to inflammation and villous atrophy. This pathological change results in a diminished surface area for nutrient absorption, contributing to systemic malnutrition. Moreover, the disruption of intestinal barrier integrity allows antigens to translocate, potentially triggering a cascade of autoimmune reactions. This immunological response is mediated by T-cells in the intestinal mucosa that react adversely to gluten, leading to the production of antibodies against transglutaminase, an enzyme involved in tissue repair [17].

Diagnosis: Challenges and Tools

Diagnosing celiac disease can be challenging due to its variable clinical presentations, which can range from typical G symptoms to atypical non-GI manifestations, such as anemia and fatigue. Enhanced awareness and improved screening strategies have facilitated earlier detection and intervention. Serologic tests, such as those for anti-tissue transglutaminase (tTG) and anti-endomysial antibodies, are typically used to identify individuals likely to have celiac disease. An intestinal biopsy remains the gold standard for confirmation, showing characteristic histopathological features like villous atrophy and crypt hyperplasia. Additionally, monitoring symptoms in response to a gluten-free diet can further support the diagnosis and assess the efficacy of dietary interventions [18,19].

Managing Celiac Disease: Gluten-Free Diet and Beyond

The primary treatment for celiac disease is a strict, lifelong gluten-free diet, which requires the elimination of all sources of gluten to manage symptoms and prevent complications. However, adhering to such a diet can be challenging due to the ubiquity of gluten in food products and the risks of cross-contamination. Emerging therapies aim to provide additional management options and improve the quality of life for patients. These include enzymatic treatments to degrade gluten peptides before they trigger an immune response, agents that strengthen intestinal tight junctions to prevent gluten peptide leakage, and immune modulators that reduce inflammatory responses to gluten. These treatments are in various stages of research and development, offering hope for less restrictive future management of celiac disease [20,21].

Associated Conditions and Complications

Celiac disease is associated with numerous complications and comorbid conditions due to its systemic effects. Nutrient malabsorption can lead to deficiencies in vitamins and minerals, contributing to anemia and osteoporosis. There is also an increased prevalence of other autoimmune disorders, such as type 1 diabetes and thyroid disease, among celiac patients. Additionally, there is a slightly elevated risk of GI cancers. Dermatitis herpetiformis, a skin manifestation of celiac disease, is characterized by itchy, blistering rashes and underscores the disease's systemic nature. Regular medical follow-ups and ongoing monitoring are crucial to manage these associated conditions and prevent severe complications [22].

The interconnected gut: understanding the gut microbiome

Role of the Microbiome in Digestive Health

The gut microbiome, an intricate ecosystem of microbes residing in our digestive tract, plays a pivotal role in human health. These microbial communities are crucial for nutrient metabolism, the synthesis of vitamins and amino acids, and the breakdown of indigestible compounds. Beyond nutritional support, the microbiome is essential in regulating immune responses, maintaining the integrity of the gut epithelium, and protecting against pathogens. The diversity and balance of microbial species within the microbiome are directly linked to overall health, with imbalances often associated with numerous diseases [23].

Microbiome and Its Impact on Gastrointestinal Disorders

Dysbiosis, or the disruption of this microbial balance, is increasingly recognized as a key factor in the development and progression of GI disorders such as IBD, IBS, and colorectal cancer. These disruptions can lead to chronic inflammation, increased intestinal permeability (leaky gut), and altered immune responses. Current research focuses on understanding the specific pathways through which these microbes influence gut health and disease, aiming to identify potential therapeutic targets. Modulating the gut microbiome through targeted interventions offers promising strategies for treating and even preventing some of these conditions [24].

Probiotics, Prebiotics, and Diet

Probiotics and prebiotics represent two strategies aimed at positively influencing the composition of the gut microbiome. Probiotics consist of live beneficial bacteria that, when administered in adequate amounts, confer a health benefit on the host. They help restore the natural balance of the gut flora, which can be disrupted by factors like antibiotics or poor diet. Prebiotics, on the other hand, are non-digestible fibers that feed the beneficial bacteria already present in the gut, promoting their growth and activity. The integration of these supplements into the diet has shown promise in managing symptoms of various GI disorders, improving digestion, and enhancing immune function.

Diet plays a fundamental role in shaping the microbiome. Dietary patterns that include diverse plant-based foods and fiber are beneficial, while diets high in processed foods and sugar can promote harmful bacteria. Transitioning from a Western diet to a more balanced, nutrient-rich diet can lead to significant improvements in microbiome health and overall wellness. However, maintaining these dietary changes can be challenging, and transitioning back to a more typical diet often leads to a reversion to the previous microbial state. Understanding these dynamics is crucial for developing sustainable dietary recommendations that promote long-term gut health [25].

Advances in gastrointestinal research and treatment

Emerging Therapies and Medications

The landscape of GI therapy is rapidly evolving with the introduction of novel medications tailored to individual physiological and genetic profiles. Gut-specific steroids and advanced biologics have revolutionized the management of inflammatory conditions by specifically targeting inflammatory pathways with minimal systemic side effects. Pharmacogenomically optimized agents, which are tailored based on a patient’s genetic makeup, offer an unprecedented level of personalized care, enhancing therapeutic efficacy and minimizing adverse reactions. Additionally, the technique of fecal microbial transplantation has moved from a niche treatment to a broader application across various GI disorders, reflecting its potential to restore microbial balance and treat conditions like recurrent *Clostridium difficile* infections. Moreover, the emerging field of viromics provides valuable insights into the viral components of the human microbiome, opening new avenues for understanding and managing GI health [26,27].

Technological Innovations in Diagnosis and Monitoring

Recent technological advancements have significantly enhanced the diagnostic and monitoring capabilities in gastroenterology. High-resolution manometry and video capsule endoscopy have become pivotal in assessing motility disorders and visualizing the small bowel with remarkable detail, respectively. These technologies facilitate a more accurate diagnosis and assessment of GI diseases, leading to more targeted treatment strategies. Furthermore, the integration of genetic testing and microbiome assays has provided deeper insights into the molecular and microbial underpinnings of GI disorders, enabling more precise and personalized therapeutic approaches. The application of biosensors and nanotechnology in this field allows for continuous, real-time health monitoring, offering a dynamic view of a patient’s condition and response to treatment, thereby optimizing management plans based on real-time data [28,29].

Future Directions in Gastrointestinal Research

The future of GI research is poised to be driven by several promising areas. Stratified medicine approaches, which involve segmenting patient populations based on genetic, biomarker, or phenotypic characteristics to tailor therapy, are gaining traction. This approach promises to enhance the efficacy and safety of treatments by aligning them more closely with the individual characteristics of patients. Research into the microbiome ecosystem and metatranscriptomic analyses are shedding light on the complex interactions within the gut microbiota and their impact on health and disease, heralding new microbial intervention strategies that could transform preventative and therapeutic modalities in gastroenterology. Additionally, the continued exploration of the effects of diet, stress, and lifestyle on gut health provides critical data that could lead to holistic, non-pharmacological management strategies for GI conditions. These areas of research not only hold the potential to uncover novel therapeutic targets but also to redefine our understanding of GI health and disease management [30].

Navigating patient care in gastrointestinal disorders

Patient Education and Lifestyle Guidance

Effective management of GI disorders extends beyond medical treatment to include significant lifestyle and dietary modifications. Educating patients about the impact of diet on symptoms is crucial; tailored advice often includes reducing intake of irritants like caffeine and spicy foods, increasing fiber, and maintaining a balanced diet to support digestive health. Stress management techniques such as meditation, yoga, and regular physical activity are recommended to mitigate stress-related exacerbation of symptoms. Additionally, patients are advised to abstain from smoking and limit alcohol consumption, as these can aggravate digestive symptoms. Providing patients with comprehensive educational materials on their condition, potential treatment side effects and strategies for symptom management empowers them to make informed healthcare decisions and actively participate in their treatment planning. Self-monitoring techniques, such as symptom diaries and trigger tracking, are invaluable tools that help patients and healthcare providers refine treatment strategies over time [31].

Psychological Aspects and Quality of Life

The psychological impact of chronic GI conditions cannot be overstated. Conditions like IBS and IBD are often associated with increased rates of anxiety and depression, which can worsen symptoms and affect overall quality of life. Recognizing these bidirectional relationships is vital for effective management. Routine psychological screening and integrated mental health services are essential components of comprehensive patient care. Providing access to psychological support through counseling and support groups helps patients cope with the challenges of their condition, enhancing their resilience and capacity to manage their health. These support structures are not just supplementary but integral to improving patient outcomes in GI healthcare [32].

Multidisciplinary Approach to Management

The complex nature of GI disorders requires a holistic and coordinated approach to care, involving a multidisciplinary team of health professionals. Gastroenterologists, dieticians, mental health professionals, physical therapists, and primary care providers must collaborate to address the multifaceted needs of these patients. This team-based approach ensures that all aspects of the patient’s health are considered, from the physical symptoms of the GI disorder to the psychological and social factors that affect illness and recovery. Regular team meetings and shared treatment plans can enhance communication between different specialties, ensuring that care is seamless and fully integrated. This comprehensive care model not only helps in managing the disease more effectively but also significantly improves the patient's overall well-being and quality of life [33].

## Conclusions

This review highlights the complexities and impacts of major GI disorders, GERD, IBD, gastritis/PUD, and celiac disease, on health and society. It emphasizes the importance of understanding the pathophysiology, diagnostic challenges, and advancements in treatment. The role of the gut microbiome and lifestyle adjustments in therapy is noted, as well as the need for a multidisciplinary approach encompassing education, lifestyle guidance, and psychological support. The bidirectional relationship between mental health and GI symptoms underscores the importance of holistic treatment. Overall, a comprehensive, patient-centered approach integrating research and collaborative efforts is crucial for improving outcomes and reducing the societal burden of GI disorders.
